# The impact of digital health technologies on women’s health nursing: opportunities and challenges

**DOI:** 10.4069/whn.2025.06.05

**Published:** 2025-06-30

**Authors:** Ju-Hee Nho

**Affiliations:** College of Nursing, Research Institute of Nursing Science, Jeonbuk National University, Jeonju, Korea

## Introduction

With technological advancements, nursing has also rapidly transitioned toward digitalization. Digital health significantly contributes to health promotion by improving health management systems and empowering individuals for self-management. In women’s health nursing specifically, digital health technologies are particularly valuable due to their advantages in providing continuous health management and enhanced accessibility for women experiencing diverse health challenges across their lifespan [1,2]. The World Health Organization (WHO) has presented a strategic roadmap aimed at achieving universal health coverage through the “Global Strategy on Digital Health 2020–2025.” This strategy is designed to enhance the health status of all individuals through accessible, appropriate, and cost-effective digital health technologies, emphasizing core principles such as equity, sustainability, safety, and person-centeredness [1].

## Digital health research in women’s health nursing

Digital health encompasses artificial intelligence, mobile health (mHealth), telemedicine, wearable devices, and electronic health records. Recently, numerous digital health studies have been published in *Women’s Health Nursing*, with various participants and topics. Notable examples include a study evaluating the quality of 163 pregnancy-related mobile apps available in Korean app stores [3], the development and user assessment of a mobile application promoting healthy lifestyle behaviors among overweight and obese breast cancer survivors [4], a topic modeling study analyzing online parenting community posts to explore parental awareness regarding neonatal metabolic screening [5], and a study investigating the influence of digital literacy on depressive symptoms among older women, including the mediating role of social support [6]. In September 2023, a special issue of *Women’s Health Nursing* titled “Digital Era Education for Women’s Health and Well-being” was published, featuring nine original articles and two editorials focused on the design, development, effectiveness, and use of digital interventions and programs supporting women’s health and well-being. This special issue included systematic reviews, scoping reviews, experimental studies, and cross-sectional studies. To commemorate its 30th anniversary as the official journal of the Korean Society of Women’s Health Nursing [7], *Women’s Health Nursing* is publishing this special issue on digital health interventions for women’s health. I hope readers find these manuscripts informative and useful. This editorial highlights strategic approaches based on advances in digital technology and evolving nursing roles, specifically addressing the health needs of women.

## Strategic approaches for women’s health nursing according to the development of digital health technology

In women’s health nursing, practices, education, and research involving digital health are rapidly evolving. To further guide this development, it is necessary to review the implications by reflecting the strategies suggested by the WHO [1].

First, it is crucial to strengthen equity in digital health for women. The WHO emphasizes reducing disparities in accessibility and use of digital health technologies as a core strategy. Women often face digital exclusion due to factors such as socioeconomic vulnerability, older age, and rural residence. Therefore, nurses caring for women must serve as mediators, facilitating improved access to digital devices and supportive environments, and enhancing digital health literacy among women.

Second, maintaining quality assurance in nursing through digital technology is essential. WHO highlights the necessity of rigorous quality management, effectiveness assessments, and safety evaluations of digital health technologies [1]. Beyond scientifically validating mobile applications and wearable technologies used in women’s health nursing, critical appraisal and participant education are essential before their clinical adoption. A relevant study indicates that health applications may mislead consumers by promoting claims without scientific backing or by presenting unproven technologies through scientific-sounding language [8]. Nurses must thus provide rigorous validation and transparent information about digital health technologies to offer users trustworthy content.

Third, achieving person-centered nursing in digital health practice is vital. WHO advises that digital health requires genuinely person-centered management rather than merely introducing new technologies [1]. Nurses should design tailored nursing interventions reflective of women’s unique health challenges across various life stages, employing digital tools that foster autonomy and active participation.

Finally, establishing clearer policies on digital health and bolstering nursing leadership accordingly is paramount. The WHO strategy recommends that countries develop policies, legislation, and regulatory frameworks for digital health, alongside enhancing healthcare professional leadership, particularly among nurses. In women’s health, nurses should move beyond clinical roles to become central stakeholders—implementers and policy collaborators—driving the introduction and expansion of digital health initiatives [9].

## Changes in the role of nurses due to the spread of digital health technology

The roles of nurses must evolve in alignment with the strategic direction of women’s health nursing, influenced by advancements in digital health technologies as discussed above. Nurses’ roles can broaden beyond clinical care to encompass being health information providers, educators, decision-makers, ethical advocates, and nursing leaders. Particularly in women’s health, which demands continuous health management across life-cycle events (e.g., pregnancy, childbirth, and menstrual problems) nurses’ digital health capabilities become increasingly crucial.

### Health information providers

Individuals acquire health information from diverse digital sources, including mobile apps, wearable devices, and web-based platforms. However, they often lack the ability to verify the accuracy, relevance, and ethical considerations of this information. Nurses should function as health information providers who guide patients toward reliable digital health information and interpret it effectively, thus facilitating personalized health management. Additionally, nurses can act as information brokers, helping bridge the gap between digital sources and individuals’ unique health needs [10].

### Educators for improving digital health literacy

Older women, those with lower educational levels, and women from socioeconomically vulnerable backgrounds frequently have limited access to digital health resources and lower health literacy. Nurses can significantly contribute by educating these women on how to effectively use smart devices, install and navigate health applications, and interpret health-related data, ultimately enhancing their digital health literacy [11,12].

### Data-based nursing decision-makers

Digital health technologies can capture extensive data, including real-time biometric measurements, health behavior patterns, and emotional responses of patients. Nurses must leverage these data to promptly identify risk factors and formulate data-driven nursing interventions. This approach facilitates precise identification of nursing problems and the creation of targeted nursing plans tailored to women’s varying health concerns throughout their life cycle.

### Ethical and legal advocates

The proliferation of digital health technologies raises ethical challenges, including breaches of privacy, algorithmic biases, and potential violations of patient autonomy. Nurses must act as ethical advocates, ensuring the use of digital technology respects patient rights and safeguards sensitive women’s health information. They must remain vigilant in protecting sensitive information and advocating for ethical standards in digital health applications [13].

### Leaders in digital nursing transformation

Nurses in clinical environments such as hospitals and community health centers should assume leadership roles in planning nursing information systems, evaluating new technologies, and educating nursing staff as central figures in digital health implementation. Nursing leaders, in particular, must guide the integration of digital technologies and nursing practices from a change-management perspective, thereby enhancing nursing quality across their organizations [9].

## Conclusion

Applying WHO’s Global Strategy on Digital Health to women’s health nursing clearly indicates that digital health technologies must evolve beyond mere tools, simultaneously promoting women’s health equity, autonomy, nursing professionalism, and ethical standards. Staying by the patient’s side and embodying a more person-centered approach in a professional manner, while being adept at adopting digital health technologies, not only expands the role of nurses but also serves as a basis for nursing to create differentiated value in the process of digital transformation of the healthcare system.
